# Association of Moderate-to-Vigorous Physical Activity, Sedentary Time, Fat Percentage, and Physical Fitness with Gait Parameters in Women with Fibromyalgia: The Al-Ándalus Project

**DOI:** 10.3390/biomedicines12040829

**Published:** 2024-04-09

**Authors:** Sergio Llorente-Romero, Manuel Herrador-Colmenero, Pedro Acosta-Manzano, Milkana Borges-Cosic, Blanca Gavilán-Carrera, Pedro Ángel Latorre Román, Manuel Delgado-Fernández, Víctor Segura-Jiménez

**Affiliations:** 1 GALENO Research Group, Department of Physical Education, Faculty of Education Sciences, University of Cádiz, 11519 Puerto Real, Spainvsegura@ibsgranada.es (V.S.-J.); 2 La Inmaculada Teacher Training Centre, Sport and Health University Research Institute (iMUDS), University of Granada, 18013 Granada, Spain; 3 Physical Activity for Health Promotion Research Group (PAHELP), Sport and Health University Research Institute (IMUDS), Department of Physical Education and Sports, Faculty of Sport Sciences, University of Granada, 18011 Granada, Spainmanueldf@ugr.es (M.D.-F.); 4 Institute of Human Movement Science, Sport and Health, University of Graz, 8010 Graz, Austria; 5 Sport and Health University Research Institute (IMUDS), University of Granada, 18007 Granada, Spain; 6 Department of Physical Education, Faculty of Education Sciences, University of Cádiz, 11519 Puerto Real, Spain; 7 Physical Activity for Health Promotion Research Group (PAHELP), 18071 Granada, Spain; 8 Departamento de Didáctica de las Lenguas, las Artes y el Deporte, Facultad de Ciencias de la Educación, Universidad de Málaga, 29010 Málaga, Spain; 9 Department of Corporal Expression, University of Jaen, Campus Las Lagunillas, s/n, 23071 Jaén, Spain; 10 Biomedical Research and Innovation Institute of Cádiz (INiBICA) Research Unit, Puerta del Mar University Hospital, 11009 Cádiz, Spain; 11 UGC Medicina Física y Rehabilitación, Hospital Universitario Virgen de las Nieves, 18013 Granada, Spain; 12 Instituto de Investigación Biosanitaria ibs.GRANADA, 18012 Granada, Spain

**Keywords:** accelerometry, aerobic capacity, exercise, gait analysis, strength

## Abstract

Gait impairments have been found in women with fibromyalgia, reducing the physical activity possibilities in this population and leading to a negative correlation with fibromyalgia impact. The aim of this study was to analyze the individual and independent associations of moderate-to-vigorous physical activity (MVPA), sedentary time, fat percentage, and physical fitness with gait parameters in women with fibromyalgia. A total of 84 women with fibromyalgia were included. MVPA and sedentary time were assessed with accelerometry, fat percentage with bioimpedance analysis, and physical fitness with field-based fitness tests. Gait was assessed during a “6 min walk test” and categorized in velocity, cadence, step length, step cycle duration, unipedal stance phase, and bipedal stance phase. Individual relationships were analyzed by partial correlations and independent relationships by linear regressions, adjusting by age and height. MVPA, sedentary time, fat percentage, and physical fitness were correlated with most gait parameters (rpartial between |0.842| and |0.219|; *p* ≤ 0.05). Physical fitness was independently associated with all gait parameters (β between |0.346| and |0.761|; *p* ≤ 0.002). In addition, MVPA was independently associated with velocity and step length (β = 0.241 and 0.292; both *p* = 0.004), and fat percentage was associated with bipedal stance phase (β = 0.242; *p* = 0.049). Good levels of MVPA, physical fitness, and adequate weight balance are associated with improved gait parameters in women with fibromyalgia.

## 1. Introduction

Fibromyalgia is mainly characterized by chronic widespread pain, fatigue, and cognitive impairments, as well as an increased risk of comorbidities [1]. Cabo-Meseguer et al. [2] have recently established the world prevalence of fibromyalgia to be 2.1% of the population, 2.3% in Europe, and 2.4% in Spain. The diagnosis and treatment of fibromyalgia is complicated due to its unknown etiology and to the heterogeneity of patients [3]. 

Gait can be considered to be one of the locomotor tasks with more clinical relevance [4]. Gait has been widely used to assess and monitor general health and functional status [5]. Gait parameters are a predictor of hospitalization and decline in general health and physical function in elderly populations [6,7], and slow walking speed is one of the criteria used to detect frailty [8]. In fact, they are also considered a predictor of falls [9,10], mortality (mediated by physical activity), and disability [11]. People with disability have shown a decreased quality of life due to the decline in the physical functions [12] causing a lack of independence in daily life activities [13], as well as facing an extra cost of living [14]. There are multiple protocols described in the available literature to analyze gait parameters. While some studies have used habitual or self-selected pace walking tests [15,16], others have used maximal speed tests [17,18] or measurements during a 6 min test [19], with most of them using different distances and instruments for the measurements. Several studies have revealed that patients with fibromyalgia showed gait impairments such as higher variability [20] (or lower regularity [21]), but also spatio-temporal [22] gait impairments such as decreased velocity, cadence, and step length, or different distributions of gait phases like decreased swing phase. One study [23] did not find any differences in spatio-temporal parameters or joint kinematic; however, they found an altered muscle synergy when walking. Góes et al. [24] have compared the gait parameters of women with fibromyalgia with those of the elderly individuals and found no significant difference between these groups in spatio-temporal parameters and the range of motion of lower-body joints, suggesting that middle-aged women with fibromyalgia have similar gait characteristics to elderly controls. Heredia et al. [22] found a negative correlation between quality of the gait parameters (velocity, stride length, and single support) and the impact of fibromyalgia. Also, Auvinet et al. [21] found a positive correlation between stride frequency and the impact of fibromyalgia.

The large number of symptoms might lead patients with fibromyalgia to be afraid of performing physical activity, therefore increasing their time in sedentary behaviors [25]. In addition, fibromyalgia patients have shown greater levels of fat percentage than those of non-fibromyalgia individuals [26] and deteriorated physical fitness, with values comparable to those of elderly populations [27]. Moreover, some of these factors have a relationship with different symptoms and outcomes, like pain [28,29,30], fatigue [29], severity [28], or cognitive function [31]. Different studies have suggested that physical activity [15,16] (especially moderate-to-vigorous physical activity-MVPA [32]), sedentary time [33], fat percentage, and physical fitness [34] might be associated with functioning in individuals with fibromyalgia, although evidence on the relationship of these variables with gait is unknown. Studying the individual and independent relationship of lifestyle-related variables like physical activity, sedentary behavior, fat percentage, and physical fitness with gait parameters would provide more evidence on the importance of behaviors and lifestyle in this population. 

Therefore, it is expected that women with fibromyalgia with greater time spent in MVPA and less in sedentary behaviors, lower body fat, and higher physical fitness present better gait parameters. The objective of the current study was to analyze the individual and independent association between MVPA, sedentary time, fat percentage, and physical fitness with kinematic gait parameters in women with fibromyalgia.

## 2. Materials and Methods

### 2.1. Study Design and Participants

Participants from this cross-sectional study belonged to the al-Ándalus project [35]. Briefly, participants from Andalusia (southern Spain) were contacted through fibromyalgia associations (via informative meetings, flyers, and e-mail) and internet advertisements (via local newspaper in online version). After the study dissemination, potential participants who contacted with the research team received verbal study information in group meetings in fibromyalgia associations, or by telephone. At the end of the group meetings or before the start of this study, participants were instructed to read the informed consent and to sign it in the case of agreement to join to the study. A total of 125 participants signed the written informed consent and agreed to participate. To be included in this study, patients had to be previously diagnosed with fibromyalgia by a rheumatologist, be a woman, and be ≤65 years old. In addition, participants were screened and needed to meet the modified 2011 American College of Rheumatology criteria [36]. Exclusion criteria were: (i) any acute or terminal illness or (ii) present severe cognitive impairment (Mini Mental State Examination (MMSE) score < 10). This study was reviewed and approved by the Ethics Committee of the “Hospital Virgen de Las Nieves” (Granada, Spain). All participants signed an informed consent after receiving information about this study.

### 2.2. Procedures

**Figure 1:**
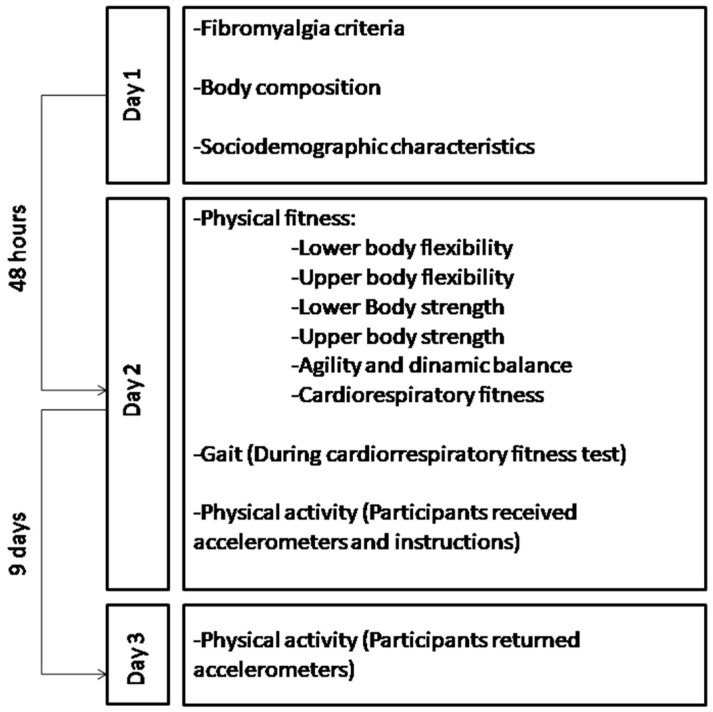
Data collection timeline and test order.

Measurements were made on two different days. Data collectors had graduated in a Sport Sciences and Biomedicine Ph.D. with extensive expertise. The measurements were performed in 4 different cities in Andalucía (Carmona, Córdoba, Málaga, and Jaén) in the south of Spain using University or fibromyalgia association facilities. On the first day, participants fulfilled the fibromyalgia criteria and filled out the different questionnaires. In addition, their body composition was assessed. On the second day, participants were asked to perform all the physical fitness tests. Additionally, participants were given the accelerometer device and were instructed on how to use it and how to fill out a sleep diary. After 9 days, participants returned the accelerometer to the research team. The data collection timeline and test order are shown in Figure 1.

### 2.3. Measurements

#### 2.3.1. Sociodemographic Characteristics

Participants self-reported their age, marital status, educational status, and current occupation. Marital status was categorized into married or not married; current occupation into working, housekeeper, or unemployed; and educational status into university degree or non-university degree.

#### 2.3.2. Inclusion and Exclusion Criteria Measurements

The modified 2011 criteria for the fibromyalgia diagnosis published by the American College of Rheumatology were used as the inclusion criteria [36]. The widespread pain index (WPI) measured the number of areas where they had pain during the previous week, divided into 5 different regions. The total score of the WPI questionnaire ranges from 0 to 19. The symptom severity scale (SSS) measured the severity of symptoms like fatigue, waking up unrefreshed, and cognitive symptoms, assigning each one a value from 0 to 3, where 3 is the most severe. In addition, the SSS considers the presence of headaches, pain, or cramps in the lower abdomen as well as depression. The total score of the SSS ranged from 0 to 12. Patients were positively diagnosed when a WPI score ≥7 and an SSS score ≥ 5 was attained, or when a WPI score between was 4–6 and an SSS score ≥ 9 was attained, with pain being present in at least 4 out of 5 regions of the body (not including the jaw, chest, and abdominal pain), and symptoms being present for at least 3 months.

The Mini Mental State Examination (MMSE) was used as an exclusion criterion. The MMSE is a questionnaire measuring five cognitive domains and it provided information about the cognitive performance and possible dementia of participants. The score ranges from 0 up to 30, and scores < 10 were considered as presenting severe dementia [37].

#### 2.3.3. Physical Activity and Sedentary Time

Physical activity was measured by using a triaxial accelerometer (GT3X+, Actigraph, Pensacola, FL, USA) during 9 consecutive days. Raw data were processed in counts per minutes, which are a measurement of the acceleration, at a rate of 30 Hz and stored at an epoch length of 60 s. The device was worn on an elastic belt placed on the waist [38], close to the center of gravity. Data from the first and the last day as well as sleeping time (reported by the participants through a sleep diary) were not included in the analysis. Therefore, 7 days with at least 10 h of wear time were required to be included in the analyses. Bouts of 0 counts during at least 90 min were considered non-wear periods [39], and excluded from analysis. Cut points were used to establish sedentary time, and the MVPA intensity levels were <200 and >2690 counts per minute (cpm), respectively [40,41]. The manufacturer’s software (ActilifeTM v.6.13.4 desktop) was used to download and process the raw data, which were presented in min/day. The percentage of total wear time spent in MVPA and sedentary time were calculated and used for analysis.

#### 2.3.4. Body Composition

Fat percentage and weight (kg) were measured by bioelectrical impedance analysis (InBody R20, Biospace, Seoul, Republic of Korea). Participants were asked not to have a shower, perform intense physical activity, eat, or drink (or consume only small amounts of fluid or food) in the previous two hours before the measurement. Moreover, during the measurement, participants wore only underwear and did not wear any metal objects. The validity and reliability of this instrument has been previously reported [42]. In addition, height (cm) was measured using a stadiometer (Seca 22) without shoes while participants were standing and with their head positioned in the Frankfort plane. Body mass index was calculated as weight divided by square height expressed in meters.

#### 2.3.5. Physical Fitness

Physical fitness was measured by using the Functional Senior Fitness Test Battery [43]. The reliability and feasibility of the tests included in the battery have been previously studied [44], and these tests have been used previously in this population. Participants were encouraged during the test performances.

Lower-body flexibility was measured by using the chair sit and reach test, where the participants started in a sitting position and tried to reach or pass their toes by bending forward sliding the hands down. The results were registered in centimeters, with negative scores indicating the participant did not reach her toes, and positive scores indicating the participant passed her toes. Participants were given two non-consecutive tries for each leg and the final score was an average of the best score from each leg.

Upper-body flexibility was measured by using the back scratch test. This test measures the distance between the middle fingers when trying to reach each other behind the back. The measure was registered in centimeters, in a negative score if fingers did not reach each other or positive if they passed. Participants were given two non-consecutive tries for each hand, and the final score was an average of the best score from each hand.

Lower-body strength was measured by using the 30 s chair stand test. The participants started the test sitting, and the goal was to completely stand up as many times as possible within 30 s.

Upper-body strength was measured by using the arm curl test, which measures the number of curls in 30 s that the participant is able to perform when lifting a weight of 2.3 kg. The total score was an average of the repetitions reached with each arm.

Agility and dynamic balance were assessed by using the 8-foot up-and-go test, where the participants were asked to stand up from a chair, walk around a cone placed at 8 ft (2.44 m), and sit back in the chair as fast as possible. The participants were told to start the test with their arms crossed over their chest (so that they could not use them to stand up) and use them normally once they started walking. The best score of two trials was registered in seconds. The values of this test were inverted, as higher scores indicated lower performance.

Cardiorespiratory fitness was assessed by using the 6 min walk test, which measures the number of meters walked by a participant along a rectangular course of 45.7 m during 6 min. The rate of perceived exertion as well as the heart rate at the end of the test and one minute after were registered.

#### 2.3.6. Gait Analysis

**Figure 2:**
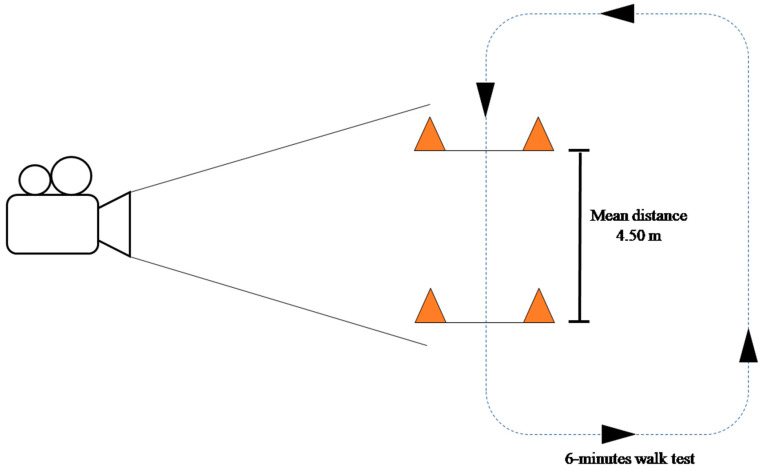
Camera set up.

Participants were recorded while performing the 6 min walk test, as it has been proven to be an appropriate test for the assessment of gait patterns in individuals with fibromyalgia [19]. The data used for the analysis were obtained as the average of the parameters from the second and the second to last lap. Sagittal video (50 Hz; 50 fps) was recorded using a high-speed camcorder (Panasonic HC-V160EC-K). The mean recording distance was 4.5 m, ranging between 4.20 and 5 m due to the characteristics of the different facilities where measurements were performed. The camera set up is shown in Figure 2. Video data were analyzed using a 2D video editor (Kinovea, Arquennes, Belgium, version 0.8.27) by individuals trained to ensure inter-rater reliability. Kinovea has previously been validated as a tool to assess time-related variables [45] and measure distances in 2D when a distance of 5 m is kept and angles of perspective go from 90° to 45° [46]. The parameters analyzed were as follows: (i) velocity (meters/seconds), measured considering the reference distance and the time needed to walk along it; (ii) cadence (step/minute), by manually counting the number of steps and dividing this by the amount of time recorded; (iii) step length (centimeters), as the mean values of both feet in the distance between the heel of the advanced foot and the toes of the back foot; (iv) step cycle duration (seconds), as the mean of both feet of the time between two strokes of the same foot; (v) unipedal stance time (seconds), as the amount of time spent standing on only one leg during a whole step cycle expressed as a mean of both feet; (vi) bipedal stance time (seconds), as the amount of time spent standing on both legs during a whole step cycle; (vii) both unipedal and bipedal stance phases (% step time) were obtained when expressing unipedal and bipedal stance time as a percentage of the step cycle.

### 2.4. Statistical Analysis

The characteristics of the participants were analyzed using descriptive statistics (mean and standard deviation for continuous variables and frequency and percentage for categorical variables). Partial correlation analyses were used to assess the individual associations of MVPA, sedentary time, fat percentage, and physical fitness with gait parameters, adjusting by age and height.

Subsequently, multivariate linear regression models were built to assess the independent associations between those variables which presented a previous correlation with the gait parameters. Given that all the physical fitness tests were correlated with at least one gait parameter, a global physical fitness variable was calculated. The scores from each test were standardized using the z-score (value − mean/SD). Global physical fitness was computed as the weighted average of all the z-scores. Enter method was used to select the included variables. Therefore, MVPA, sedentary time, fat percentage, and global physical fitness were included simultaneously as independent variables and gait parameters were included as dependent variables. The model was adjusted by age and height.

Normality, linearity, and homoscedasticity were tested by using normal probability plots of the standardized residual and scatterplots of residuals. No serious multicollinearity problems among the predictor variables of the model were found (all variance inflation factor statistics ≤ 2.2). Statistical significance was set at *p* < 0.05 for all analyses, which were performed using the Statistical Package for Social Sciences IBM-SPSS version 22.0 for Windows.

## 3. Results

A total of 41 participants were excluded from the analysis as 11 were not previously diagnosed, 8 were men, 4 were older than 65 years old, and 7 did not meet the new fibromyalgia criteria. Additionally, 11 participants presented missing data in some of the study variables. The final sample included 84 participants whose characteristics are shown in Table 1.

Concerning individual associations, partial correlations between the variables of the study are shown in Table 2. MVPA and sedentary time were correlated with velocity (r_partial_ = 0.31 and −0.29, respectively, all *p* < 0.01) and step length (r_partial_ = 0.38 and −0.34, respectively, all *p* < 0.01). In addition, sedentary time was inversely associated with unipedal stance phase (r_partial_ = −0.29, *p* < 0.01) and positively associated with bipedal stance phase (r_partial_ = 0.28, *p* < 0.05). Fat percentage was negatively associated with velocity (r_partial_ = −0.32), step length (r_partial_ = −0.34), and unipedal stance phase (r_partial_ = −0.35), and positively associated with bipedal stance phase (r_partial_ = 0.37), (all *p ≤* 0.01). Most physical fitness components were positively associated with velocity, cadence, step length, and unipedal stance phase (r_partial_ between 0.29 and 0.85, all *p* < 0.05), except for upper-body flexibility with velocity and cadence, and lower- and upper-body flexibility and upper-body strength with unipedal stance phase (all, *p* > 0.05). All the physical fitness components were negatively associated with step cycle duration and bipedal stance phase (r_partial_ between −0.22 and −0.75, all *p* < 0.05), except for the association of step time with both flexibility measurements and upper-body strength, and the association of bipedal stance with lower-body flexibility and upper-body strength. Additionally, global physical fitness was positively associated with velocity, cadence, step length, and unipedal stance phase (r_partial_ between 0.44 and 0.79, all *p* < 0.01), and negatively correlated with step cycle duration and bipedal stance phase (r_partial_ between −0.43 and −0.69, all *p* < 0.01). 

Concerning the independent associations, results from linear regressions are shown in Table 3. MVPA was independently associated with velocity (β = 0.241, *p* = 0.004) and step length (β = 0.241, *p* < 0.001), while sedentary time was not independently associated with any gait parameter. Fat percentage was independently associated with bipedal stance phase (β = 0.242, *p* = 0.048). Moreover, global physical fitness was independently associated with velocity (β = 0.217), cadence (β = 0.656), step length (β = 0.482), step cycle duration (β = −0.700), unipedal stance phase (β = 0.369), and bipedal stance phase (β = −0.346), (all *p* ≤ 0.002).

## 4. Discussion

The objective of the present study was to provide evidence of the associations of MVPA, sedentary time, fat percentage, and physical fitness with gait parameters in women with fibromyalgia. The results suggest that greater MVPA and fitness, and lower fat percentage and sedentary time, are overall individually associated with better gait patterns. Among the different physical fitness tests, cardiorespiratory fitness presented the strongest correlations with gait parameters, followed by dynamic agility and lower-body strength. Physical fitness was independently associated with every gait parameter, while MVPA was only independently associated with velocity and step length, and fat percentage was independently associated with bipedal stance phase.

From the different protocols available to analyze gait parameters, the 6 min walk test was used in the current study [19]. As expected, our sample walking pace was faster than the self-selected paces but slower than those measured at maximal speed since the 6 min walk test is not a usual pace test nor a maximal speed test. The individuals from the current study showed worse gait parameter values than the ones found by Heredia et al. [19] in the same population. This could be explained by the higher mean age and weight found in our study sample.

Regarding research on the association of physical activity with gait parameters, Izawa et al. [17] found a positive association between MVPA and gait speed, but not with light-intensity physical activity in elders. Furthermore, there is evidence of the association of lower MVPA with reduced gait speed, step length, step time, and increased cadence in older women [47]. A randomized controlled trial [32] studied the effect of physical activity on gait speed in older adults and found that engaging in vigorous physical activity improved gait parameters, suggesting that changes can only be detected if the lower limb muscles are sufficiently stimulated. On the other hand, several studies have found an association between sedentary time and gait speed [48,49] among older adults, but this was a small association and not robust [49] after adjusting for MVPA. Moreover, a recent study explored the relationship between sedentary time and gait speed, and it was discovered that physical fitness could have a mediating role between them [50]. Therefore, intensity could be a requisite to detecting changes in gait parameters, and our results appear to be consistent with previous research, showing a small association of sedentary time with gait parameters that disappeared in the regression model when MVPA, fat percentage, and physical fitness were considered. Consequently, participating in MVPA might improve gait given the independent association of MVPA with velocity and stride length found in the current study.

Previous research has shown that the greater the BMI status is, the worse impact on the kinematics and kinetics parameters of gait is [51]. It has been theorized that adaptations in the obese population might reduce the moment of force in the knee and the energy expenditure per unit time, or could be a way to maintain balance [52]. Of importance, the mean body mass index of the participants in the current study was lower than that of the aforementioned study (28.9 vs. 33.1); however, we found similar correlations between variables. In another study in an elderly population, Latorre-Román et al. [18] found a negative correlation between fat percentage and gait speed in a 10 m maximal speed test, which agreed with the results of the present study. Nonetheless, it is important to bear in mind that the associations between fat percentage and gait parameters disappeared when sedentary time, MVPA, and physical fitness were considered, except for bipedal stance phase. Therefore, our results support that individual associations of fat percentage and gait parameters observed in the literature might be mainly moderated by individuals’ MVPA and/or physical fitness levels. 

Physical fitness showed a consistent and independent association with gait parameters in the current study. Furthermore, the inclusion of diverse physical fitness components let us know that some of them presented greater associations with gait than others. Cardiorespiratory fitness showed the strongest associations, followed by dynamic agility, probably since both measures are assessed through walking behaviors. Contrarily, strength and flexibility showed the weakest associations with gait. A cross-sectional study in older adults [53] presented very similar results to those presented in the current study, showing that fitness (measured with the senior fitness test battery) presented stronger associations with gait parameters than body mass index or self-reported physical activity, especially walking endurance. Although we cannot assume the causality of the association due to the cross-sectional design of this study, it has been hypothesized that improving cardiorespiratory fitness might improve gait parameters through a restructuring in the central nervous system or by activating cortical areas involved in walking such as the prefrontal cortex [54]. This might be explained by the fact that improving cardiorespiratory fitness typically involves some form of walking, running, or any cyclic movement of the lower limbs that could potentially improve gait pattern. However, if the cardiorespiratory training consisted of upper-body movements alone, this relationship might not exist. A previous study in adults with intellectual disabilities [55] who also faced gait impairment found lower strength to be correlated with poorer spatio-temporal gait parameters. Gait was measured at a fast pace and strength was measured by the “30 s chair stand test” and “manual dynamometry test”, which is comparable with the methods used in the current study. However, when measuring gait at the comfortable speed, only velocity was positively associated with a higher score in the “30 s chair stand test”. It has been hypothesized that strength improvements can improve gait speed through biomechanical factors, with the strength of the hip extensors and ankle plantar flexors acting as the best predictors of gait speed improvement [56]. However, there is no clear evidence of the effect of stretching on gait performance in older adults [57]. Also, in the current study, an unexpected relationship between upper-body strength and flexibility with multiple gait parameters was found. A possible explanation might be that these factors can contribute to general coordination and balance, but also that individuals with better lower-body strength and flexibility usually present better upper-body scores (correlation coefficient between lower- and upper-body flexibility in the current study was 0.28, *p* = 0.009). Since previous studies have not found associations between upper-body flexibility and gait parameters, future studies are encouraged to confirm the associations observed in the current study.

Improving gait parameters might be relevant for patients with fibromyalgia, not only because of fall prevention and disability [9,10,11] but also because of its relationship with a lower impact of fibromyalgia [20,21]. Moreover, better gait parameters are related to functional independence in daily life activities [34], which could potentially improve the quality of life and facilitate social relationships. Knowing the association of gait parameters with MVPA, sedentary time, fat percentage, and physical fitness in women with fibromyalgia, their assessment could be a useful methodology to estimate the functional independence of this population. Additionally, future experimental research is needed to ascertain whether exercise programs, that might lead to physical fitness improvements and fat percentage reductions, along with MVPA promotion, could influence gait parameters in these patients, and whether these changes would be beneficial for patients.

The current study presented some limitations: (i) the cross-sectional design did not allow us to establish causality, which is necessary to corroborate in future intervention studies of the causal associations between these variables; (ii) the sample was chosen by convenience, increasing the risk of bias. On the other hand, the strengths of our study are: (i) this is the first study of the independent association of sedentary time, MVPA, fat percentage, and fitness with gait parameters in women with fibromyalgia; (ii) the objective measurement of physical activity and sedentary time, which is usually self-reported via questionnaires; (iii) the measurement of physical fitness was assessed by a reliable battery of tests in this population.

## 5. Conclusions

MVPA, sedentary time, fat percentage, and physical fitness are individually associated with several gait parameters. However, physical fitness was the only variable which showed an association with all gait parameters, independently of MVPA, sedentary time, and fat percentage. The results seem to encourage women with fibromyalgia to exercise by focusing on improving physical fitness, which is usually accompanied by MVPA promotion and fat percentage reductions. These relationships should be further studied through experimental research.

## Figures and Tables

**Table 1:** Characteristics of the participants (*n* = 84).

Variable	Mean	SD
Age (years old)	53.5	7.2
Weight (kg)	71.8	15.7
Height (cm)	157.5	6.2
WPI (score)	14.7	3.0
SSS (score)	7.8	1.9
BMI (kg/m^2^)	28.9	5.9
MVPA (wear time %)	4.8	3.6
Sedentary time (wear time %)	51.3	11.0
Body fat (%)	40.3	7.6
Physical fitness		
Lower-body flexibility (cm)	−11.5	10.4
Upper-body flexibility (cm)	−14.5	10.5
Lower-body strength (repetitions)	11.4	3.3
Agility and dynamic balance (s)	−6.4	1.3
Upper-body strength (repetitions)	16.4	4.9
Cardiorespiratory fitness (m)	520.2	67.3
Gait		
Velocity (m/s)	1.6	0.2
Cadence (step/s)	132.8	12.3
Step length (cm)	44.2	6.2
Step cycle duration (s)	0.5	0.0
Unipedal stance (s)	0.4	0.0
Bipedal stance (s)	0.1	0.0
Unipedal stance phase (% of step time)	79.6	4.5
Bipedal stance phase (% of step time)	20.5	4.5
	**n**	**%**
Marital Status		
Married	64.0	76.2
Unmarried	20.0	23.8
Educational status		
Non-universitary	73.0	86.9
Universitary	11.0	13.1
Current occupational status		
Working	19.0	22.6
Housekeeper	24.0	28.6
Unemployed	41.0	48.8

BMI = Body mass index; MVPA = Moderate-to-vigorous physical activity; SD = Standard deviation; SSS = Symptom severity score; WPI = Widespread pain index.

**Table 2:** Pearson’s correlations between gait parameters and physical activity, sedentary time, fat percentage, and physical fitness (n = 84).

Outcomes	Gait Parameters					
	Velocity (m/s)	Cadence (Step/min)	Step Length (cm)	Step Cycle Duration (s)	Unipedal Stance Phase (% Step Cycle)	Bipedal Stance Phase (% Step Cycle)
MVPA	0.31 **	0.11	0.38 **	−0.12	0.16	−0.14
Sedentary time	−0.29 **	−0.18	−0.34 **	0.16	−0.29 **	0.28 *
Fat percentage	−0.32 **	−0.16	−0.34 **	0.12	−0.35 **	0.37 **
Lower-body flexibility	0.40 **	0.35 **	0.35 **	−0.35 **	0.07	−0.06
Upper-body flexibility	0.16	−0.01	0.29 **	−0.02	0.21	−0.22 *
Lower-body strength	0.57 **	0.55 **	0.31 **	−0.55 **	0.25 *	−0.23 *
Agility and dynamic balance	0.64 **	0.52 **	0.46 **	−0.57 **	0.40 **	−0.37 **
Upper-body strength	0.60 **	0.54 **	0.38 **	−0.53 **	0.13	−0.13
Cardiorespiratory fitness	0.85 **	0.72 **	0.64 **	−0.75 **	0.60 **	−0.59 **
Global PF	0.79 **	0.66 **	0.59 **	−0.69 **	0.44 **	−0.43 **

MVPA = moderate-to-vigorous physical activity; PF = physical fitness. Adjusted by age and height. * = *p* < 0.05; ** = *p* < 0.01.

**Table 3:** Linear regression models assessing the independent association of moderate-to-vigorous physical activity, sedentary time, fat percentage, and global fitness with gait parameters (n = 84).

Models	B	95% CI	β	*p*
Velocity (m/s)				
MVPA (% of wear time)	0.01	(0.00, 0.02)	0.24	0.004
Sedentary time (% of wear time)	0.00	(−0.00, 0.01)	0.07	0.438
Body Fat (%)	−0.00	(−0.01, 0.00)	−0.08	0.301
Global PF (z-score)	0.22	(0.18, 0.26)	0.76	<0.001
Cadence (step/min)				
MVPA (% of wear time)	0.05	(−0.64, 0.74)	0.01	0.888
Sedentary time (% of wear time)	−0.03	(−0.30, 0.23)	−0.03	0.809
Body Fat (%)	0.11	(−0.21, 0.42)	0.07	0.509
Global PF (z-score)	10.70	(7.81, 13.60)	0.66	<0.001
Step length (cm)				
MVPA (% of wear time)	0.50	(0.17, 0.83)	0.29	0.004
Sedentary time (% of wear time)	0.01	(−0.12, 0.14)	0.02	0.866
Body Fat (%)	−0.12	(−0.27, 0.03)	−0.15	0.120
Global PF (z-score)	3.95	(2.57, 5.33)	0.48	<0.001
Step cycle duration (s)				
MVPA (% of wear time)	0.00	(−0.00, 0.00)	−0.03	0.734
Sedentary time (% of wear time)	0.00	(−0.00, 0.00)	0.00	0.990
Body Fat (%)	−0.00	(−0.00, 0.00)	−0.11	0.254
Global PF (z-score)	−0.04	(−0.05, −0.03)	−0.70	<0.001
Unipedal stance phase (% of Step cycle)				
MVPA (% of wear time)	0.06	(−0.25, 0.36)	0.05	0.705
Sedentary time (% of wear time)	−0.04	(−0.16, 0.08)	−0.09	0.527
Body Fat (%)	−0.12	(−0.26, 0.02)	−0.20	0.098
Global PF (z-score)	2.18	(0.90, 3.46)	0.37	0.001
Bipedal stance phase (% of Step cycle)				
MVPA (% of wear time)	−0.03	(−0.34, 0.27)	−0.03	0.824
Sedentary time (% of wear time)	0.04	(−0.08, 0.15)	0.09	0.557
Body Fat (%)	0.14	(0.00, 0.28)	0.24	0.049
Global PF (z-score)	−2.05	(−3.34, −0.76)	−0.35	0.002

B = non standardized regression coefficient; CI = confidence interval: β = standardized regression coefficient; MVPA = moderate-to-vigorous physical activity; PF = physical fitness. Linear regression models were built using gait parameters as dependent variables and MVPA, sedentary time, fat percentage, and global PF as independent variables (enter method). All models were adjusted for age and height.

## Data Availability

Data are contained within the article.
